# Growing Up in a Changing Climate: How Temperature Affects the Development of Morphological, Behavioral and Physiological Traits of a Marsupial Mammal

**DOI:** 10.3389/fphys.2020.00049

**Published:** 2020-02-12

**Authors:** Clare Stawski, Fritz Geiser

**Affiliations:** ^1^Centre for Behavioural and Physiological Ecology, Zoology, University of New England, Armidale, NSW, Australia; ^2^Department of Biology, Norwegian University of Science and Technology, Trondheim, Norway

**Keywords:** activity, antechinus, body mass, endotherm, metabolic rate, phenotypic plasticity

## Abstract

Climate change is likely to affect many mammalian phenotypes, yet little is known whether and how phenotypic plasticity is involved in responding to thermal challenges during mammalian development. We investigated the effect of continuous cold or warm exposure during development on morphological, behavioral, and functional variables of yellow-footed antechinus (*Antechinus flavipes*), a semelparous Australian marsupial mammal. Captive-bred young were exposed to two ambient temperatures (T_*a*_), cold (17°C) or warm (25°C), once weaned. Treatments were reversed and metabolic rate (MR) measurements repeated after 2 months. We measured body mass weekly, activity continuously, and MRs over a range of T_*a*_ once they were adults. Growth rate was similar in both groups, but was faster in males. Antechinus in the warm group were initially more active than the cold group and decreased activity when exposed to cold, whereas the cold group increased activity when exposed to warm. Interestingly, females changed their night-time activity when T_*a*_ was changed, whereas males changed their daytime activity. MRs were originally lower in the warm group in comparison to the cold group for both sexes and increased slightly for females, but not for males, after being exposed to cold. After exposure to warm T_*a*_, the MRs of the cold group decreased significantly over the entire T_*a*_-range for both sexes. Our results reveal that temperatures experienced during development can influence behavioral and physiological traits in antechinus. Such phenotypic plasticity is vital for a species that within 1 year is dependent on a single breeding event and experiences a complete population turnover.

## Introduction

Ambient temperature (T_a_) is known to influence critical developmental stages of many organisms. Traits affected by T_a_ during development range from body size to sex to energetics and, importantly, adult phenotypes are a result of the environment in which the individual developed (Scharloo, 1989; Pigliucci et al., 1996). An increase in mean T_a_ due to climate change is likely to substantially affect organism development. However, little is known about how species respond morphologically, behaviorally and physiologically during development to new thermal challenges and such data are particularly lacking for endotherms (Williams et al., 2008; Huey et al., 2012).

Temperature effects on growth rates in ectotherms are relatively well understood. In general, most ectotherms grow more slowly under cold conditions in comparison to warm conditions (Angilletta, 2004). Conversely, how different T_a_ affect growth rates during development in endotherms is poorly understood, although for many organisms cold environments can often select for larger body sizes, known as Bergmann’s rule (Bergmann, 1847; Meiri and Dayan, 2003; Angilletta, 2004). Growth rate is vital in species that have a short period in which to become mature in order to either reproduce before perishing or to survive their first winter period. For example, species that inhabit extreme arctic and alpine environments have a protracted active season in which to gather enough fat reserves or food to survive the long winter (Morrison et al., 1954; Bieber et al., 2017). Importantly, T_a_ has an immediate effect on the activity patterns of many endotherms. Most notably, many species of birds and mammals reduce activity levels as T_a_ decreases (Vickery and Bider, 1981; Brice et al., 2002; Ashdown and McKechnie, 2008), because remaining active is energetically costly at colder T_a_. Therefore, activity patterns in many endotherms change seasonally, such that they display more activity during the warmer summer months. It may be that the activity level of an individual during development as a result of the T_a_ can impact not only on their adult activity levels, but also on their resulting growth rates and metabolic rates (MRs).

Understanding an animal’s MR is vital to interpreting how the individual functions in its current environment and how it may adapt to new conditions. In particular, MR governs both day-to-day energetic requirements and more long-term variables such as growth rate and survival (Anderson and Jetz, 2005; Burton et al., 2011). Further, mechanisms that are employed to balance energy loss and gain on a daily basis must be under strong selection pressure, as being in a negative energy state is highly undesirable (Auer et al., 2016; Stawski et al., 2017a). In order to maintain a high and stable body temperature (T_b_), endotherms have much higher and adjustable MR in comparison to ectotherms. This allows endotherms to be active largely independent of environmental T_a_. However, both cold and hot T_a_ increase MR in order to produce heat or remove heat, respectively. This relationship of MR with T_a_ is best represented by the thermoregulatory curve, which also encompasses the thermoneutral zone (TNZ). The TNZ is the range of T_a_ where the animal does not need to thermoregulate and can display basal metabolic rates (BMR). Below the TNZ, the MR must increase to compensate for heat loss, above the TNZ it increases to facilitate heat loss via evaporative cooling. The thermoregulatory curve provides a useful tool to determine how environmental variables affect the energetics of endothermic animals when at rest. For example, many endotherms display a seasonal shift of the thermoregulatory curve in order to acclimate to varying environmental conditions, such as a change in weather and photoperiod, throughout the year (Zhao et al., 2010; Bozinovic et al., 2011; Boratyński et al., 2016). However, even more advantageous than flexibility in response to predictable seasonal changes, is phenotypic flexibility in response to unpredictable changes (Riek and Geiser, 2012; Boratyński et al., 2016). which would be advantageous to adapt to climatic changes.

The aim of our study is to provide quantitative data on the effect of continuous cold and warm exposure during development on morphological and functional variables of the marsupial antechinus. These insectivorous marsupials have a most unusual life history. In the wild they breed once a year over a short 2-week period (Naylor et al., 2008). The mating period is followed by a complete male die-off at about 11 months of age, attributed to a combination of stress-related factors (Naylor et al., 2008). Females, on the other hand, must survive at least for 15 months until their large litters of young are weaned and a few females may live for a second year (Naylor et al., 2008; Rojas et al., 2014). Importantly, apart from these females, there is only a single cohort of individuals at any given time. This type of life history pattern, known as semelparity, is rare in mammals and typically only in those that inhabit predictable seasonal environments as their mating period usually commences based on changes in photoperiod (Naylor et al., 2008). Because antechinus exhibit a complete population turnover in a single year they offer an ideal model for investigating effects of T_a_ on morphological and functional variables to gain a better understanding on how animal populations are likely to respond in the short term to thermal challenges they will experience during climate change. We hypothesized that cold-rearing and warm-rearing will affect morphology, behavior and physiology irreversibly. Importantly, changes in activity and MR are likely to be correlated in order to cope with the varying energetic demands created by the different thermal treatments. If observed, such phenotypic responses are important for coping with different climates over a wide range of distribution, but also play a crucial role for enhancing survival during climate change.

## Materials and Methods

All procedures were approved by the University of New England Animal Ethics committee.

**FIGURE 1:**
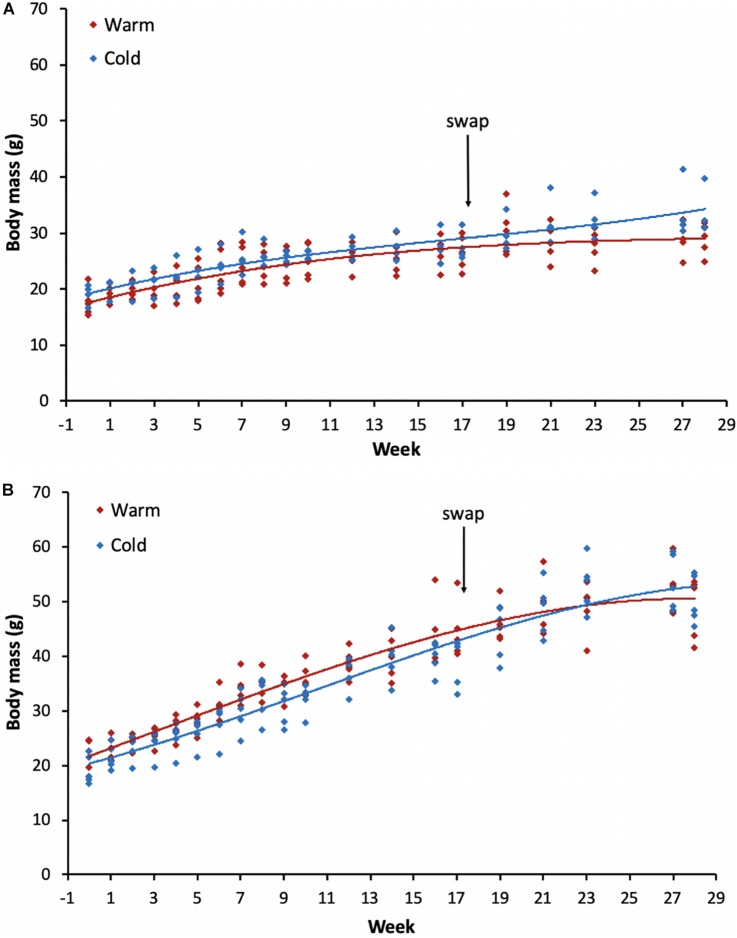
Body mass changes for all individuals post-weaning (from ∼100 days old) throughout the experiment for **(A)** females and **(B)** males for each of the treatment groups. The arrow indicates the week when the acclimation temperatures were reversed for the groups. Red lines indicate the group that was initially placed in the warm treatment and the blue lines the group in the cold treatment. Week 0 represents body mass on the day the individuals were placed into the temperature controlled rooms.

Initially, eight yellow-footed antechinus (*Antechinus flavipes*, Marsupialia, Dasyuridae; five females, three males) were captured in the wild from Aberbaldie Nature Reserve (31°04′24′′S, 151°25′34′′E) in Australia and studied in a different experiment (see Stawski et al., 2017b). After the conclusion of this previous experiment (end of July) these wild-caught adults were held together in a large outdoor aviary with access to several nest boxes placed at various heights. They were supplied with *ad libitum* food and water. From July to October the reproductive status of these adults was monitored and to ensure successful reproduction, in mid-August all individuals were transferred to an indoor facility and kept in individual cages (see Stawski and Rojas, 2016). The females gave birth to a total of 19 young (9 females, 10 males) from early to mid-September. Once the young were weaned [early December at ∼ 100 days of age (see Westman et al., 2002)], they were separated from their mothers and placed into individual cages (week 0 on Figure 1). These juveniles were then randomly split into two treatment groups, warm or cold, and placed into rooms at a T_a_ of 16.7 ± 1.3°C (*n* = 199 days; *N* = 4119 measurements; cold) or 24.7 ± 1.3°C (*n* = 199 days; *N* = 4119 measurements; warm). Body mass (± 0.1 g) of all individuals was measured with an electronic balance once a week throughout the duration of the experiment.

Activity patterns of all of the juvenile antechinus was measured throughout the experiment with passive infrared sensors attached to individual cage lids and recorded with custom-made data loggers (Körtner and Geiser, 1995). The sensors pick up movements and these were summed on the data loggers over 10-min periods. Sensors were placed facing directly down into the cage of the animal, which prevented the sensors from picking up any movement from animals in nearby cages. Short time periods (∼5 min) when experimenters were feeding and replacing water in the cages or longer time periods (∼60 min) when animals were being weighed and the cages cleaned, were noted and these data were removed from analyses.

When the juvenile antechinus reached adult size (end of March at ∼220 days of age), the thermal response of MR as a function of T_a_ was quantified for each individual. MR was measured as rate of oxygen consumption (V̇O_2_) and was obtained via open-flow respirometry. A linear mass loss throughout measurements was obtained by weighing the antechinus before and after each measurement and this was used for calculation of mass-specific V̇O_2_ values. A FOX analyser and a FC-1B analyser (both from Sable Systems International, Las Vegas, NV, United States, resolution 0.001%) were used to obtain the V̇O_2_ measurements. The FOX analyser system switched in sequence between three animal channels and a reference channel every 3 min, whereas the FC-1B analyser system switched between two animal channels and a reference channel every 3 min. Mean V̇O_2_ values were derived from periods when oxygen consumption was minimal and stable for >3 consecutive readings (i.e., over > 27 min). Rotameters (Aalborg, NY, United States) were used to control the rate of airflow into the chambers and mass flow meters (Omega FMA-5606, Stamford, CT, United States) were used to measure the rate of airflow. A flow rate of 500 ml O_2_ min^–1^ was used for each animal chamber and 200 ml O_2_ min^–1^ for the analyser subsample. Voltage outputs from flow meters and oxygen analysers were recorded on a computer via a 14-bit A/D converter card. Air samples were dried using silica gel. Glass jars (1000 ml) were used as respirometry chambers to house the antechinus during measurements. Once the antechinus were in these chambers they were placed within a temperature-controlled unit. T_a_ within the temperature-controlled unit and in each of the individual chambers was measured at the same time as V̇O_2_ readings by a calibrated thermocouple (±0.1°C, Omega DP116 digital thermocouple thermometer, Stamford, United States); these data were transferred to the computer via the A/D converter card. V̇O_2_ was calculated by using equation 3a of Withers (Withers, 1977) and G. Körtner wrote the data acquisition program.

Two experimental protocols for the MR measurements were used. For both protocols the animals were measured during their daytime resting period to ensure resting MR was obtained. For the first protocol the animals were placed into the chambers at T_a_ 18°C and the T_a_ was increased in 4°C increments every 2 h up to 30°C. The T_a_ began at 14°C for the second protocol and was reduced to 10°C after 3 h. The exact T_a_ of each chamber varied; therefore the individual chamber T_a_ were used when analyzing the data.

At the end of the first round of MR measurements acclimation T_a_ were reversed (at ∼230 days of age). After the reversal, weekly body mass measurements were continued and activity was again measured continuously with the passive infrared sensors. After 3 months of acclimation (at ∼320 days of age) the MR measurements were repeated as described above. This was to ensure that measured functional variables did not simply reflect temperature acclimation rather than developmental phenotypic adjustments.

The two treatment groups (cold and warm) were split into the following four groups for analysis to account for the reversal treatment:

• Cold: individuals that were initially housed under cold conditions.
• Cold-Warm: individuals that were initially housed under cold conditions and then transferred to warm conditions.
• Warm: individuals that were initially housed under warm conditions.
• Warm-Cold: individuals that were initially housed under warm conditions and then transferred to cold conditions.

All data analyses were conducted using “R” Studio (R Development Core Team, 2009). To test what factors (date, body mass, sex, treatment, T_a_) affected the measured variables (body mass, whole day activity, daytime activity, night activity, resting MR, basal metabolic rate) linear mixed effects models were fitted (function “lmer” in package “lme4”) and ranked using AIC (function “dredge” in package “MuMIn”). To account for repeated measures, individuals were included as a random effect. Once the top model had been selected for each of the measured variables, a Shapiro test was run on the residuals to ensure normal distribution, which was the case for all of the variables. Afterward, a Tukey test (function “lsmeans” in package “lsmeans”) was run to see what treatment groups differed significantly from each other (*p* < 0.05).

## Results

### Growth

The best-fit model revealed that growth rates among sexes and treatment groups were described by date, sex, treatment and an interaction between sex and treatment (Table 1). As expected, individuals increased body mass with time throughout the experiment across both sexes and all treatment groups (Figure 1). No significant differences were found between the treatment groups for the same sex (*p*-values ranged from 0.100 to 1.000). However, there was a significant difference between sexes (*p*-values ranged from <0.0001 to 0.035), such that males were typically heavier than females. Starting body mass for females in the warm treatment was 17.7 ± 2.2 g (*n* = 5) and in the cold treatment was 19.1 ± 1.5 g (*n* = 4), whereas for males was 21.9 ± 2.7 g (*n* = 5) in the warm treatment and 19.2 ± 2.4 g (*n* = 5) in the cold treatment. The final body mass recorded during the entire experiment for females in the warm-cold treatment was 28.9 ± 2.6 g (*n* = 5) and in the cold-warm treatment was 33.5 ± 3.7 g (*n* = 4), whereas for males was 48.9 ± 5.2 g (*n* = 5) in the warm-cold treatment and 50.3 ± 4.0 g (*n* = 5) in the cold-warm treatment.

**TABLE 1:** The four best-fit models for each of the measured variables.

Variable	Model	AICc	Delta
BM	**date + sex + treatment + sex:treatment**	**12797.9**	**0.0**
	date + sex + treatment	15214.0	2416.1
	date + treatment	15239.5	2441.5
	date + sex	15257.9	2459.9
WDA	**bm + sex + treatment + bm:sex + bm:treatment + sex:treatment**	**52961.3**	**0.0**
	bm + sex + treatment + bm:treatment + sex:treatment	53004.3	42.9
	bm + sex + treatment + bm:sex + bm:treatment	53045.9	84.7
	bm + sex + treatment + bm:sex + sex:treatment	53094.5	133.2
DA	**bm + sex + treatment + bm:sex + bm:treatment + sex:treatment**	**49834.4**	**0.0**
	bm + sex + treatment + bm:treatment + sex:treatment	49884.9	50.5
	bm + sex + treatment + bm:sex + sex:treatment	49980.4	146.0
	bm + sex + treatment + bm:sex + bm:treatment	50017.2	182.8
NA	**bm + sex + treatment + bm:sex + bm:treatment + sex:treatment**	**51251.9**	**0.0**
	bm + sex + treatment + bm:treatment + sex:treatment	51292.8	40.8
	bm + sex + treatment + bm:sex + sex:treatment	51300.3	48.4
	bm + sex + treatment + sex:treatment	51334.8	82.9
RMR	**sex + T_a_ + treatment + sex:T_a_ + sex:treatment + T_a_:treatment**	−**99.2**	**0.0**
	sex + T_a_ + treatment + sex:T_a_ + sex:treatment	–91.4	7.8
	sex + T_a_ + treatment + sex:T_a_ + T_a_:treatment	–90.1	9.1
	sex + T_a_ + treatment + sex:treatment + T_a_:treatment	–86.8	12.4
BMR	**sex + treatment**	−**57.7**	**0.0**
	sex + T_a_ + treatment	–47.5	10.2
	treatment	–45.9	11.7
	sex + T_a_ + treatment + sex:T_a_	–39.4	18.3

*Body mass (g, BM); whole day activity (counts, WDA); daytime activity (counts, DA); night-time activity (counts, NA); resting metabolic rates (V̇O_2_, ml g^–1^ h^–1^, RMR); basal metabolic rates (V̇O_2_, ml g^–1^ h^–1^, BMR). The best-fit model for each variable is indicated in bold.*

### Activity

For all of the activity variables (whole day, day, night) the top model showed that they were influenced by body mass, sex, treatment and the interactions between these variables (Table 1). For both sexes and all treatment groups, activity levels increased as body mass increased.

**FIGURE 2:**
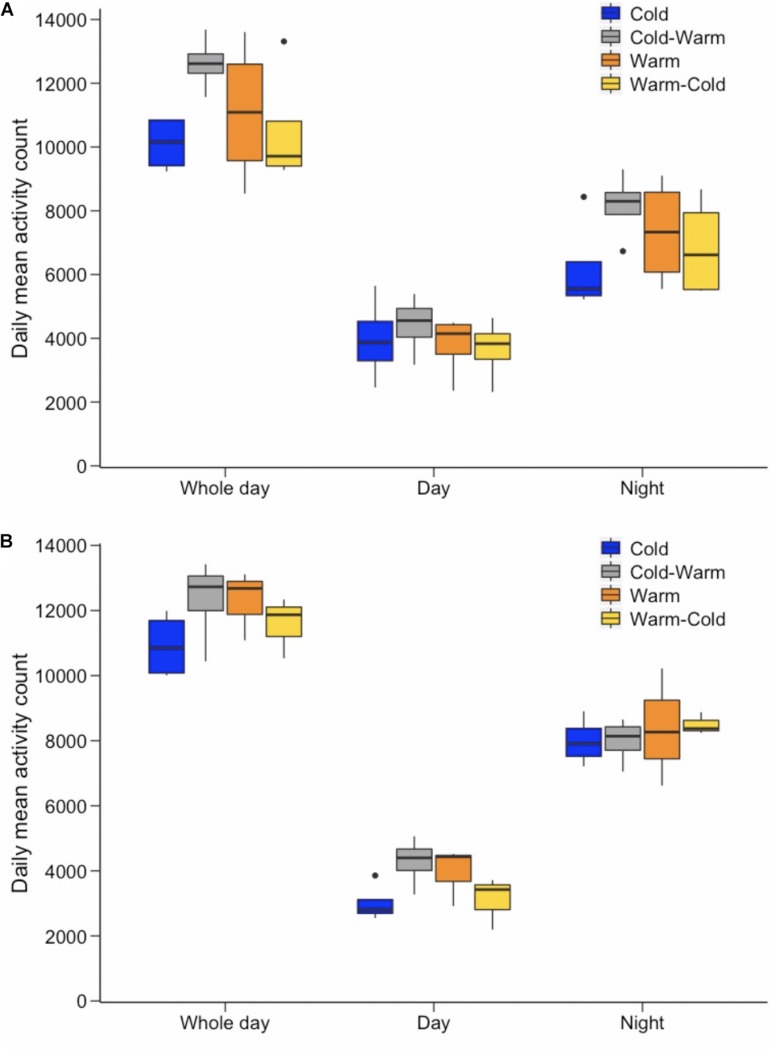
Mean activity count for **(A)** females and **(B)** males for each of the treatment groups (represented by different colors as shown in the key). Whole day, daytime, and night-time activity are presented separately.

No significant differences were found in whole day activity levels between the sexes and also between treatment groups (Figure 2; *p*-values ranged from 0.100 to 1.000). The only significant differences for whole day activity were found for females and males after individuals had been transferred to the new treatment conditions. Both males and females increased activity when transferred from the cold treatment to the warm treatment at age ∼230 days (Figure 2; females: *p*-value = 0.001; males: *p*-value = 0.009). The reverse was found when antechinus were moved from the warm to the cold treatment (Figure 2; females: *p*-value < 0.0001; males: *p*-value = 0.003).

Similar results were found for daytime and nightly activity, such that there were no significant differences among sexes and treatment groups (Figure 2; *p*-values ranged from 0.262 to 1.000). Only males displayed a significant increase in daytime activity levels when transferred from the cold to warm treatment (Figure 2B; *p*-value < 0.0001) and a decrease from the warm to cold treatment (Figure 2B; *p*-value < 0.0001). While males in both treatment groups did not alter nightly activity levels after being transferred to the new treatment at age ∼230 days (Figure 2B; *p*-values ranged from 0.643 to 1.000), females increased nightly activity when moved from the cold to the warm treatment (Figure 2A; *p*-value = 0.001) and decreased nightly activity from the warm to the cold treatment (Figure 2A; *p*-value = 0.001).

### Metabolism

**FIGURE 3:**
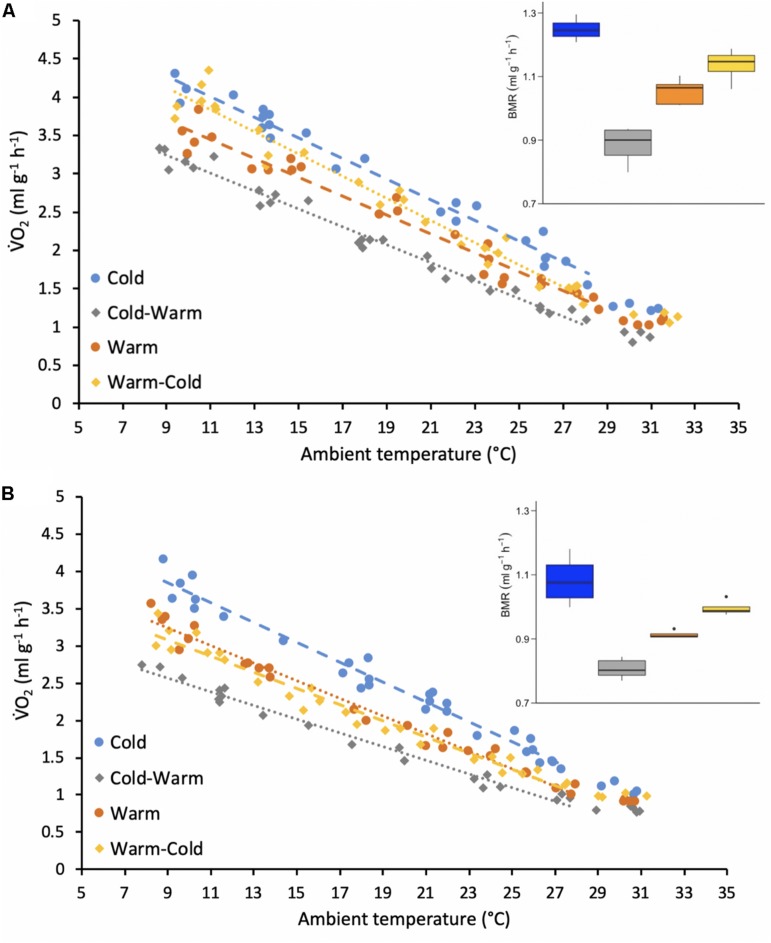
Resting metabolic rates (RMR measured V̇O_2_, ml g^–1^ h^–1^) of adult **(A)** female and **(B)** male individual antechinus at different ambient temperatures (°C). Each of the treatment groups are represented by a different color as shown by the key. The circles and dashed lines represent the initial treatment groups and the diamonds and the dotted lines represent the treatment groups after reversal. The points forming the regression lines represent resting metabolic rates (RMR) and the points without a line represent basal metabolic rates (BMR). The inset boxplots represent the BMR (ml g^–1^ h^–1^) measured for each treatment group and sex.

Mass-specific resting metabolic rates (RMR) throughout the thermoregulatory curve were best explained by sex, T_a_, treatment and interactions between these variables (Table 1). In general, RMR increased with decreasing T_a_ for both sexes and all treatment groups (Figure 3). For all treatment groups, females displayed higher RMR than males (Figure 3; *p*-values ranged from <0.0001 to 0.032). For both females and males, RMR was higher in the cold treatment group in comparison to the warm treatment group (Figure 3; all *p*-values < 0.0001) and RMR decreased when individuals were transferred from the cold treatment to the warm treatment (Figure 3; all *p*-values < 0.0001). Only for females, RMR significantly increased after individuals were transferred from the warm treatment to the cold treatment (Figure 3A; *p*-value < 0.0001). In contrast, there was no significant change in RMR after males were placed into the cold treatment after the warm treatment (Figure 3B; *p*-value = 0.635).

Only the variables sex and treatment were included in the best-fit model for BMR (Table 1). Similarly to RMR, females (warm = 1.05 ± 0.04 ml g^–1^ h^–1^, body mass = 26.4 ± 2.7 g, *n* = 4; cold = 1.25 ± 0.03 ml g^–1^ h^–1^, body mass = 27.6 ± 2.9 g, *n* = 4; warm-cold = 1.14 ± 0.05 ml g^–1^ h^–1^, body mass = 28.4 ± 2.9 g, *n* = 4; cold-warm = 0.88 ± 0.06 ml g^–1^ h^–1^, body mass = 33.1 ± 4.4 g, *n* = 4) had higher BMR for all treatments in comparison to males (warm = 0.91 ± 0.01 ml g^–1^ h^–1^, body mass = 42.7 ± 2.8 g, *n* = 4; cold = 1.08 ± 0.07 ml g^–1^ h^–1^, body mass = 47.9 ± 3.2 g, *n* = 4; warm-cold = 0.99 ± 0.02 ml g^–1^ h^–1^, body mass = 40.0 ± 6.2 g, *n* = 4; cold-warm = 0.81 ± 0.03 ml g^–1^ h^–1^, body mass = 51.7 ± 4.5 g, *n* = 4; Figure 3; all *p*-values = 0.0006). Further, for both sexes BMR was greater in the cold treatment group in comparison to the warm treatment group (Figure 3; *p*-values < 0.0001 for females and males) and decreased when individuals were transferred to the warm treatment (Figure 3; *p*-values < 0.0001 for females and males). However, BMR did not change for individuals of both sexes from the warm treatment group after acclimatization to the cold treatment (Figure 3; *p*-values ranged from 0.239 to 0.277).

## Discussion

Rearing yellow-footed antechinus under different thermal conditions resulted in changes in morphology and function and the strongest effects were seen in their behavior and physiology. Warmer temperatures resulted in greater daily activity levels and lower MRs. Such changes are typical for many mammals as seasons shift from winter to summer. However, our data suggest that at least physiologically, individuals raised in warm conditions may have less phenotypic flexibility in regard to changing temperature regimes. Our results also revealed that growth rates in antechinus are not strongly influenced by temperature, but perhaps more by sex and food availability.

Displaying sexual dimorphism, males are typically larger for most antechinus species (Naylor et al., 2008), which can also be seen in the juvenile growth rates in the current study. Similarly to a previous study on brown antechinus (*Antechinus stuartii*; Westman et al., 2002), the yellow-footed antechinus in our study showed a steeper linear increase in body mass initially, which flattened out as they aged producing a sigmoidal growth curve. However, males continued to increase body mass more significantly than females throughout their life. In relation to acclimation temperatures and reversal, body mass for either male or female antechinus was not affected. In contrast, in accordance with Bergmann’s rule, some birds and mammals display higher body mass after cold acclimation in captivity and also during winter in the wild in comparison to summer (Meiri and Dayan, 2003; Zhang and Wang, 2007; Zhu et al., 2012; Wu et al., 2015; Hu et al., 2017). Such increases in body mass as a result of cold acclimation may be due to an increase in food intake (Hu et al., 2017). As the amount of food that the antechinus in our study consumed did not change throughout the experiment and was the same for both treatment groups, they may not have had the means to significantly alter their body mass. However, some other mammals also display no changes in body mass when acclimated to warm and cold environments. and instead increase their thermogenic capacity rather than body mass to deal with colder temperatures (Steffen and Roberts, 1977; Wang et al., 2006). The approaches of increasing body mass and/or increasing thermogenic capacity to colder conditions are only feasible for animals with diets that permit continual feeding throughout the year. For insectivorous species such as antechinus, this is not possible as their main food source is greatly reduced in the cold. Extensive reviews on Bergmann’s rule have found mixed support for temperature being the main factor influencing body size in mammals, and have suggested that other factors such as food availability are more important (Alhajeri and Steppan, 2016; Gohli and Voje, 2016). As antechinus are known to increase torpor use when exposed to cold conditions to save energy (Geiser, 1988; Parker et al., 2019), it is likely that they increased torpor use when exposed to the cold treatment. Therefore, this strategy, along with changes in activity patterns, may reduce body mass loss under cold conditions and explain why there was no difference between the treatment groups in our study.

Warmer temperatures typically lead to increased activity levels in endotherms (Vickery and Bider, 1981), which was the general observation for the antechinus in the current study. Further, antechinus did alter their activity patterns after being placed into a new temperature regime, becoming more active in the warm and less active in the cold treatment. There is often a threshold T_a_ above which the energetic cost of activity becomes prohibitively expensive. Therefore, many endotherms will reduce activity and remain resting at not only extreme cold temperatures, but also at extreme hot temperatures (Randall and Thiessen, 1980). One way animals can avoid extremely high T_a_ is by being more active at night when temperatures are cooler. Greater nocturnal activity in both sexes is already typical for antechinus and they are often regarded as one of many nocturnal marsupials, although recent studies have revealed extensive daytime activity as well (Stawski et al., 2016). This phenotypic flexibility found for activity patterns for antechinus may be beneficial under future climate scenarios. Such changes in activity patterns in response to variation in T_a_ also often coincide with shifts in an individual’s daily energy management strategies, particularly metabolism.

The differences found in both RMR and BMR between the cold and warm treatment groups reveal that even small temperature differences during development can have long-lasting effects on an individual’s physiology. As mass-specific RMR has been shown to decrease as young mature until they reach adult size (Holloway and Geiser, 2000), it is important to note that antechinus have already developed physiological thermoregulation before they have been weaned (Westman et al., 2002). Therefore, as we measured metabolism in adult antechinus, there should be no contribution from development on the measured MR. Importantly, variation in the level of RMR can impact both fitness and survival during development and throughout the life of an organism. For example, a low RMR decreases the amount of energy an animal needs and will be optimal for survival when foraging returns are limited and/or foraging is risky (Larivée et al., 2010). However, a high RMR can also be advantageous in order to have more energy for activities such as mating and to increase cold tolerance and thermogenic capacity (Liknes and Swanson, 1996; Stawski et al., 2017a). Therefore, it would be beneficial if animals are able to adjust RMR in response to climate change and also more generally throughout the year in order to cope with prevailing weather and food conditions.

For many endotherms and the antechinus in our study, a reduction in overall T_a_ as a result of winter conditions or acclimation experiments often results in an increase in BMR (Haim et al., 1991; McDevitt and Speakman, 1994; Liknes and Swanson, 1996; Corp et al., 1997; Anderson and Jetz, 2005; McKechnie et al., 2007; Zhao et al., 2010, 2014; Bozinovic et al., 2011; Chi and Wang, 2011; Boratyński et al., 2016). This solution provides a way for endotherms to use less energy for thermoregulation in the colder winter months and to enhance their cold tolerance, as they need to use more energy once the temperature drops below the TNZ and food availability is typically reduced in cold conditions. Such changes in thermal physiology are particularly important for those species that do not alter their insulation and/or body mass in response to changing seasons (Haim et al., 1991), such as the antechinus in the current study. Further, the antechinus perhaps compensated for having higher RMR and BMR under cold conditions by having lower activity levels. On the other hand, moderately warmer temperatures require either less heat production or the employment of heat loss mechanisms such as evaporation (Anderson and Jetz, 2005; Zhao et al., 2010). Therefore, a low BMR in this case may be more beneficial, as displayed by the warm-acclimated antechinus in the current study. Although green ringtail possums can store heat at high temperatures, likely to avoid evaporative cooling (Krockenberger et al., 2012), T_b_ cannot continually increase as this would result in death, and heat storage is not an effective option for small species like antechinus. Therefore, while this strategy would be suitable for heat waves lasting several hours, it would not suffice during the longer heat waves that are already occurring. The physiological flexibility of shifting to a lower BMR and RMR may instead provide the means to cope with long-term increases in T_a_.

## Conclusion

As a shift in photoperiod historically coincided with a regular change in season and environmental conditions, it provided a reliable signal for a seasonal phenotypical change. However, this is unlikely to be the case with the increasing unpredictability of weather conditions. Therefore, while some species will be able to adapt to a changing climate by altering the timing of their reproductive and subsequent development periods (Ozgul et al., 2010; Hoffmann and Sgrò, 2011), species such as the antechinus in our study seem to be unable to do so as the timing of their reproductive period is based on photoperiod, a fixed environmental variable, rather than changes in the weather. Consequently, it is important that each generation is able to adapt to a changing environment via alterations of phenotypic traits. For a short-lived species such as antechinus, there could also be selection to flexibly adjust these traits during their developmental period, which they appear capable of for at least activity and energetics. Interestingly, it appears that the cold-acclimated rather than warm-acclimated individuals have more phenotypic flexibility in regard to RMR and BMR. As adult antechinus, especially males, spend most of their life in winter conditions it is perhaps beneficial for them to adapt more readily to changes during winter rather than summer. The capability of altering the metabolic curve for thermoregulation in response to a change in climate will be particularly advantageous.

## Data Availability Statement

The datasets generated for this study are available on request to the corresponding author.

## Ethics Statement

The animal study was reviewed and approved by the Animal Ethics Committee of University of New England.

## Author Contributions

CS and FG conceived the project and wrote the manuscript. CS collected and analyzed the data.

## Conflict of Interest

The authors declare that the research was conducted in the absence of any commercial or financial relationships that could be construed as a potential conflict of interest.
